# CircRNAs in Alzheimer's disease: What are the prospects?

**DOI:** 10.1016/j.ncrna.2023.11.011

**Published:** 2023-11-26

**Authors:** Ozal Beylerli, Aferin Beilerli, Tatiana Ilyasova, Alina Shumadalova, Huaizhang Shi, Albert Sufianov

**Affiliations:** a Central Research Laboratory, Bashkir State Medical University, Ufa, Republic of Bashkortostan, 3 Lenin Street, 450008, Russia; b Department of Obstetrics and Gynecology, Tyumen State Medical University, 54 Odesskaya Street, 625023, Tyumen, Russia; c Department of Internal Diseases, Bashkir State Medical University, Ufa, Republic of Bashkortostan, 450008, Russia; d Department of General Chemistry, Bashkir State Medical University, Ufa, Republic of Bashkortostan, 3 Lenin Street, 450008, Russia; e Department of Neurosurgery, First Affiliated Hospital of Harbin Medical University, Harbin, Heilongjiang, China; f Educational and Scientific Institute of Neurosurgery, Рeoples’ Friendship University of Russia (RUDN University), Moscow, Russia; g Department of Neurosurgery, Sechenov First Moscow State Medical University (Sechenov University), Moscow, Russia

**Keywords:** Non-coding RNAs, Circular RNAs, Alzheimer's disease, Neuroinflammation, Pathogenesis

## Abstract

Circular RNAs (circRNAs) is a fascinating covalently closed circular non-coding RNA that is abundantly present in the transcriptome of eukaryotic cells. Its versatile nature allows it to participate in a multitude of pathological and physiological processes within the organism. One of its crucial functions is acting as a microRNA sponge, modulating protein transcription levels, and forming interactions with essential RNA-binding proteins. Remarkably, circRNAs demonstrates a specific enrichment in various vital areas of the brain, including the cortex, hippocampus, white matter, and photoreceptor neurons, particularly in aging organisms. This intriguing characteristic has led scientists to explore its potential as a significant biological marker of neurodegeneration, offering promising insights into neurodegenerative diseases like Alzheimer's disease (AD). In AD, there has been an interesting observation of elevated levels of circRNAs in both peripheral blood and synaptic terminals of affected individuals. This intriguing finding raises the possibility that circRNAs may have a central role in the initiation and progression of AD. Notably, different categories of circRNAs, including HDAC9, HOMER1, Cwc27, Tulp4, and PTK2, have been implicated in driving the pathological changes associated with AD through diverse mechanisms. For instance, these circRNAs have been demonstrated to contribute to the accumulation of beta-amyloid, which is a hallmark characteristic of AD. Additionally, these circRNAs contribute to the excessive phosphorylation of tau protein, a phenomenon associated with neurofibrillary tangles, further exacerbating the disease. Moreover, they are involved in aggravating neuroinflammation, which is known to play a critical role in AD's pathogenesis. Lastly, these circRNAs can cause mitochondrial dysfunction, disrupting cellular energy production and leading to cognitive impairment. As researchers delve deeper into the intricate workings of circRNAs, they hope to unlock its full potential as a diagnostic tool and therapeutic target for neurodegenerative disorders, paving the way for innovative treatments and better management of such devastating conditions.

## 1 Introduction

Alzheimer's disease (AD) looms as an increasingly formidable challenge, relentlessly progressing as a neurodegenerative disorder that primarily afflicts the elderly population. The clinical portrait it paints is one of enduring memory deficits, cognitive impairment, and profound personality changes [1]. Yet, the true intricacy of AD unfolds at the neuropathological level, where senile plaques, neurofibrillary tangles (NFTs), and pervasive neuronal loss converge to create a devastating landscape within critical brain regions such as the hippocampus, neocortex, amygdala, and basal nucleus of Meynert [2]. Senile plaques become reservoirs for aggregated amyloid β (Aβ) proteins, born from the cleavage of amyloid precursor protein (APP) by the β-secretase and γ-secretase enzymes [3]. The relentless accumulation of Aβ aggregates not only triggers neuroinflammation through microglial activation but also exacerbates oxidative stress in neurons, ultimately culminating in intracellular calcium overload, and consequently, neuronal damage and cognitive dysfunction [4]. NFTs, in contrast, are composed of deposits of phosphorylated microtubule-associated protein Tau, predominantly localized in pre- and postsynaptic regions, neuronal axons, and even the cerebrospinal fluid [5]. The hyperphosphorylation of Tau undermines its capacity to bind to microtubules, initiating the aggregation of NFTs [6]. An early event in AD pathogenesis revolves around the delicate equilibrium between Tau phosphorylation and dephosphorylation [6].

During this complex narrative, circular RNAs (circRNAs) emerge as a novel and captivating dimension. CircRNAs epitomize a unique class of non-coding RNAs, distinguished by their covalently closed loop structure, which imparts upon them an unparalleled resistance to degradation by exonucleases [7]. Originally relegated as splicing errors, circRNAs took center stage when their multifaceted and indispensable functions came to light [8]. The advent of advanced RNA sequencing technologies has further illuminated the dynamic expression patterns and functional significance of circRNAs, transcending the realms of normal physiological processes to venture into various pathophysiological conditions, including neurological disorders [9]. CircRNAs have now elevated themselves to promising candidates, offering insight into the labyrinthine pathogenesis of AD [10,11].

In this comprehensive review, our foremost aim is to synthesize the current body of research, unveiling the role of circRNAs in AD pathology. We will embark on a journey deep into the biogenesis and multifaceted functions of circRNAs, deciphering their intricate involvement in the complex mosaic of AD. Furthermore, we will explore the profound implications of circRNAs in the early diagnosis and innovative treatment strategies for AD, casting a spotlight on their potential as invaluable biomarkers and therapeutic targets in the relentless pursuit of effective interventions for this debilitating neurodegenerative disease. Our exploration extends into the captivating realm of circRNAs, as we traverse uncharted territory to unearth their potential as pivotal players, shedding light on their capacity to decipher and ultimately combat AD.

## 2 Relationship between AD and circRNAs

### 2.1 CircRNA histone deacetylase 9

**Fig. 1:**
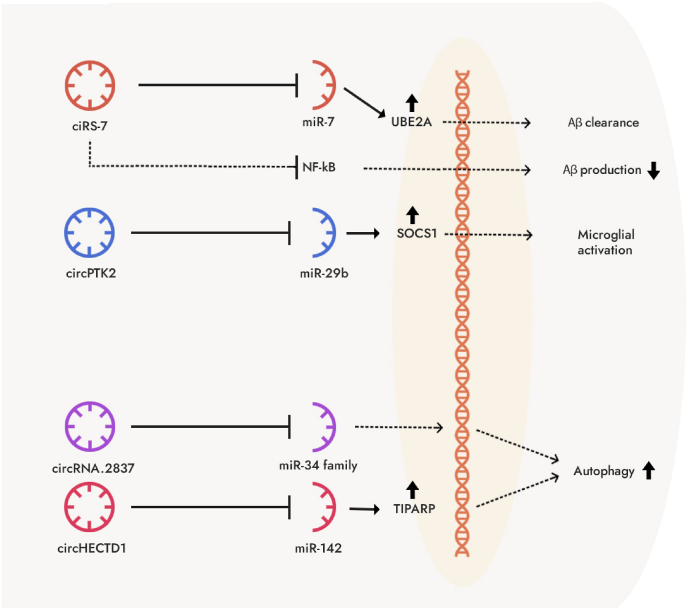
Provides a detailed schematic representation of the molecular mechanisms involving circRNAs in Alzheimer's disease (AD). a) ciRS-7 plays a significant role in regulating Aβ (beta-amyloid) clearance and production through two distinct pathways. Firstly, it acts as a sponge for miR-7, thereby relieving its suppression of UBE2A, a gene involved in Aβ clearance. Secondly, ciRS-7 influences the NF-κB pathway, affecting Aβ production. b) circPTK2 serves as a miR-29b sponge, resulting in increased expression of SOCS1, a protein linked to OGD (oxygen-glucose deprivation)-mediated microglial activation. This activation of microglia can exacerbate neuroinflammation in AD. c) The circRNAs, circRNA.2837, and circHECTD1, are involved in inducing autophagy, a cellular process crucial for maintaining neuronal health. They function as sponges for miR34 family and miR142/TIPARP axis, respectively, leading to the upregulation of autophagy-related pathways.

Synaptic dysfunction and abnormal processing of amyloid precursor protein (APP) are early pathological features of Alzheimer's disease (AD). The persistent disruption of synaptic plasticity initially manifests as a reduction in the number of synapses, which later progresses to neuronal loss. Therefore, it is speculated that synaptic loss is the basis for memory impairment in AD patients. Histone deacetylase 9 (HDAC9) is located on human chromosome 7p21 and is a member of the IIa class HDAC family. It is most abundant in the brain, skeletal muscle, heart, endothelial cells of large arteries, and smooth muscle cells [12]. It is highly expressed in the cytoplasm, and can itself cause histone deacetylation, thereby reshaping chromatin structure and controlling gene expression [13]. It plays an important role in neurological disorders and various types of tumors [14,15]. Lu et al. confirmed that the levels of circRNA HDAC9 (circHDAC9) were decreased in the serum of AD patients and patients with mild cognitive impairment [13]. Through Morris water maze experiments and Golgi staining, they found that 2-month-old APP/PS1 mice had significantly decreased spatial learning/memory abilities compared to the control group, and overexpression of miR-138 inhibited the expression of synaptic protein-I, postsynaptic density-93 (PSD-93), and PSD-95, resulting in synaptic damage (Fig. 1).

**Fig. 2:**
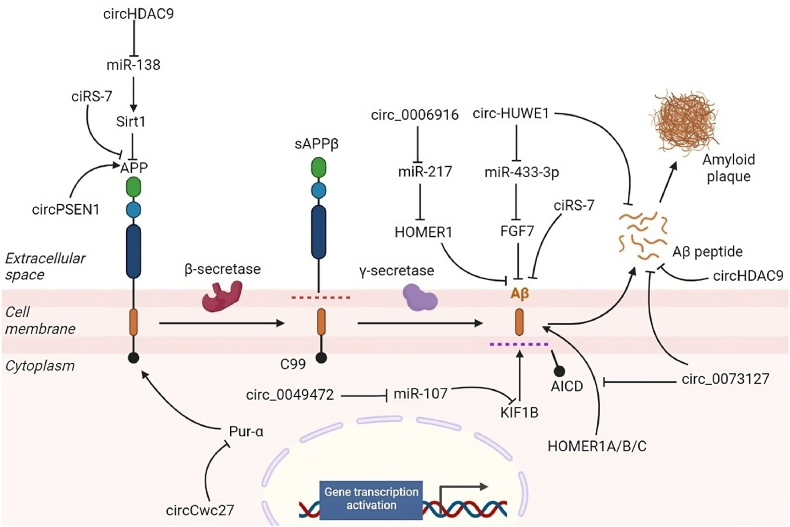
CircRNAs and their regulation amyloid precursor protein (APP) expression and amyloid plaque formation in Alzheimer's disease (AD). CircRNAs orchestrate a variety of signaling pathways involved in AD pathogenesis, including those involved in amyloid-β (Aβ) peptides production and clearance, such as expression APP. Note: KIF1B, Kinesin family member 1B; FGF7, Fibroblast growth factor 7; Pur-a, *Homo sapiens* purine rich element binding protein A.

Further analysis revealed that circHDAC9 acts as a “sponge” for miR-138 in vitro, inhibiting the expression of miR-138 and relieving AD-like phenotypes, including abnormal processing of APP, spatial learning/memory decline, and dendritic spine degeneration (Fig. 2).

Zhang et al. used real-time PCR to detect the levels of circHDAC9 and miR-142–5p and found that β-amyloid 1–42 (Aβ42) can significantly downregulate circHDAC9 and upregulate miR-142–5p in human neuronal cells [16]. Aβ42 can induce neuronal cell damage, reduce cell viability, promote cell apoptosis, and enhance inflammatory response. Subsequently, luciferase assay, RNA immunoprecipitation, and RNA knockdown experiments showed that circHDAC9 can act as a “sponge” for miR-142–5p. Overexpression of circHDAC9 exerts its “sponge” function by binding to miR-142–5p, which can alleviate Aβ42-induced neurotoxicity in HN cells. Treatment with berberine significantly increased cell viability and reduced apoptosis and inflammatory response in Aβ42-induced neuronal cells. Berberine has a protective effect against Aβ42-induced neurotoxicity and may be a potential therapeutic agent for AD. In conclusion, circHDAC9 participates in the pathological and physiological processes of AD through different signaling pathways, making it a potential biomarker and therapeutic target for AD.

### 2.2 CircRNA Homer1

Homer1 is a scaffold protein encoded, transcribed, and translated by the immediate early gene that participates in the construction of the central nervous system PSD. Homer1 is mainly divided into two types of proteins, Homer1a and Homer1b/c. Homer1a protein belongs to immediate early gene expression and is hardly expressed under physiological conditions. It is rapidly expressed in neurons after cell damage or stimulation, while Homer1b/c protein belongs to constitutive protein and is regularly expressed in the body [17,18].

CircRNA Homer1 (CircHomer1) is mainly present in the neuronal soma and dendrites and is also highly expressed in the hippocampus [18]. It plays a role in various diseases, including colorectal cancer, hepatocellular carcinoma, depression, bipolar disorder and schizophrenia, and neurodegenerative diseases [[19], [20], [21], [22]]. Studies have shown that circHomer1 in different regions of the brain is downregulated in patients with Alzheimer's disease [[23], [24], [25]].

Studies have shown that circHomer1 is downregulated in different brain regions of AD patients [[23], [24], [25]]. Network co-expression and microRNA binding site prediction analysis showed that circHomer1 was significantly associated with three studied traits, including AD patients, Braak staging of neurofibrillary tangles, and clinical dementia rating (CDR). You et al. found that circHOMER1-a, transcribed from linear RNA Homer1, was the most significantly upregulated circRNA in synaptic plasticity [18]. Li et al. showed that downregulation of circHomer1 could improve methamphetamine-induced neurotoxicity [26]. Zimmerman et al. reported that deletion of circHomer1 in the orbitofrontal cortex resulted in differential expression of mRNA subtypes related to synaptic function and psychiatric disorders, and the differentially expressed mRNA subtypes were involved in long-term inhibition of synaptic transmission, synaptic plasticity, neuronal excitation, and pre-pulse inhibition [22]. Urdánoz-Casado et al. reported that the expression of linear RNA Homer1 and circHomer1 (hsa_circ_0073,127, hsa_circ_0006916) was downregulated in the olfactory cortex of female AD patients, positively correlated with the expression of Homer1b/c protein, and negatively correlated with Aβ load, indicating that circHomer1 may play an important role in the early development of AD [27]. Cervera-Carles et al. found that circHomer1 expression was decreased in the frontal cortex of AD patients, and its expression level was negatively correlated with the pathological staging of AD (Braak staging, r = −0.178, P < 0.05) [24]. These findings suggest that circHomer1 can serve as a biomarker for synaptic pathology and an important indicator for assessing the severity of AD and may be a potential drug target for AD treatment in the future.

### 2.3 CircRNA Cwc27

CircRNA Cwc27 (CircCwc27) is a circular RNA that is highly expressed in the brain, particularly in the cortex and hippocampus, which are the brain regions most susceptible to damage in Alzheimer's disease (AD) [28]. This circRNA is enriched in neurons and shows high expression levels in the cytoplasm. It is significantly upregulated in the brains of AD patients and in mouse models of AD, while its expression in other organs such as the heart, liver, spleen, lungs, and kidneys is relatively low [28].

The Purine-Rich Binding Protein A (Pur-α) is a multifunctional RNA-binding protein that plays a key role in gene transcriptional regulation and is involved in brain development, synaptic plasticity, and memory retention [29]. Barbe et al. found that mice with Pur-α heterozygosity exhibit memory deficits, and immunohistochemical analysis of the hippocampal CA1-3 regions showed a decrease in the overall number of neurons, as well as in the number of Pur-α-immunoreactive neurons and dendrites [30].

Previous studies have shown that the expression of circRNAs in the brain is generally age-dependent [31]. However, Song et al. found that CircCwc27 levels in the hippocampus of wild-type mice slightly increased from 3 to 12 months of age [28]. Furthermore, CircCwc27 began to increase significantly three months before Aβ deposition, and its expression was significantly upregulated with age in APP/PS1 mice.

Recent research has shown that CircCwc27 directly binds to Pur-α, increasing its retention in the cytoplasm and inhibiting its recruitment to AD gene clusters, which include APP, dopamine receptor D1 (DRD1), recombinant protein phosphatase 1 regulatory subunit 1B (PPP1R1B), neurotrophic receptor tyrosine kinase 1 (NTRK1), and LIM homeobox 8 (Lhx8) [28]. The downregulation of CircCwc27 enhances the affinity of Pur-α to these promoters, leading to changes in the transcription of Pur-α target genes, suggesting that Pur-α is an important downstream mediator of CircCwc27-regulated gene expression. Overexpression of Pur-α is largely a result of CircCwc27 knockout, which prevents Aβ deposition, cognitive decline, reduces glial cell activation and pro-inflammatory cytokine production, showing a protective effect against the neuroinflammatory and neurodegenerative changes of AD. These studies indicate that the new regulatory axis composed of CircCwc27 and Pur-α may play a critical role in AD, and CircCwc27 may become a new therapeutic target for AD, and an effective tool for early diagnosis and predicting patient outcomes.

### 2.4 CircRNA Tulp4

Neuronal degeneration and synaptic changes are considered the major neurobiological basis of cognitive impairment in Alzheimer's disease (AD). Tubby-like proteins (Tulps) in vertebrates, including Tub and Tulp1-4, are thought to play important roles in this process. Tub and Tulp1-3 are closely related, while Tulp4 is more distantly related. Human Tub and Tulp1-3 are encoded by 12–15 exons, totaling 442–561 amino acids and spanning 12–15 kb, while Tulp4 is encoded by 14 exons, totaling 1543 amino acids and spanning approximately 200 kb [32]. The circRNA Tulp4 (circTulp4) in humans and mice is transcribed from the second exon of the Tulp4 gene. Rybak-Wolf et al. found that circTulp4 is highly expressed in synaptosomes in the mammalian brain and is upregulated during neuronal differentiation [33]. At the same time, circTulp4 is also abundantly expressed in the hearts of humans and mice, but not in the hearts of rats [34].

Wu et al. demonstrated that circTulp4, highly expressed in diabetes model pancreatic β cells, can regulate β cell proliferation through the miR-7222–3p/soat1/cyclin D1 signaling pathway [35]. Chen et al. found that decreased levels of circTulp4 resulted in downregulation of miR-204–5p and miR-26a-5p targets, including the MEIS2 gene, cadherin 2 (CDH2) gene, melanogenesis associated transcription factor (MITF) gene, and phosphodiesterase-4B (PDE4B) gene, affecting the development and function of the retina [36]. CircTulp4 may also be involved in AD development. For example, Ma et al. used RNA sequencing (RNA-Seq) to screen for differentially expressed circRNA sequences in APP/PS1 mice compared to wild-type (WT) mice and identified circTulp4 as a potential AD biomarker [37]. Real-time PCR results showed that circTulp4 was expressed in the brain tissue of both 2–12-month-old APP/PS1 and WT mice [37]. The expression of circTulp4 was lower in the brains of APP/PS1 mice than in those of WT mice at 9 and 12 months. Through bioinformatics analysis, chromatin isolation by RNA purification, rapid prediction of RNA-protein interactions, and chromatin immunoprecipitation, it was found that circTulp4 mainly localizes in the nucleus and interacts with U1 small nuclear ribonucleoprotein (U1 snRNP) and RNA polymerase II to regulate the transcription of its parent gene Tulp4, which regulates neuronal growth and differentiation, thereby affecting the function of the nervous system and AD development [37]. This regulatory function of circTulp4 may be a potential link between circTulp4 dysregulation and the pathogenesis of AD. Currently, there is limited research on the relationship between circTulp4 and the pathogenesis of AD, and further studies are needed to determine whether circTulp4 can be detected in body fluids, making it a new biomarker or therapeutic target for AD.

### 2.5 CircRNA proteintyrosine kinase

Proteintyrosine kinase (PTK) is a type of kinase that catalyzes the transfer of the γ-phosphate of ATP to tyrosine residues on substrate proteins, playing an important role in cell growth, proliferation, and differentiation. Changes in PTK and its phosphorylation have been found in the brains of AD patients [38]. PTK2 gene is expressed in various circRNA forms, such as hsa_circ_0003221, hsa_circ_0008305, hsa_circ_0005273, hsa_circ_0006421, and hsa_circ_0005982, all of which originate from the same PTK2 mRNA precursor but have different sequences [[39], [40], [41], [42], [43], [44], [45], [46], [47], [48]].

CircRNA PTK (circPTK2) is involved in the development of many diseases, such as non-small cell lung cancer, bladder cancer, hepatocellular carcinoma, colorectal cancer, ovarian cancer, gastric cancer, multiple myeloma, acute myeloid leukemia, tumor-associated cachexia, laryngeal squamous cell carcinoma, and glioma [49]. Currently, research on circRNA neuroinflammation in vivo and in vitro has demonstrated that neuroinflammation plays a role in the pathogenesis of Alzheimer's disease (AD) [50]. The presence of Aβ and mutations in genes encoding innate immune molecules make microglia more susceptible to stimulation and/or promote their activation, leading to the continuous production of inflammatory cytokines and chemokines, ultimately resulting in neurodegenerative changes and neuronal loss [51].

In recent years, studies have found that the downregulation of miR-137, miR-181c, miR-9, and other microRNAs can lead to the development of Alzheimer's disease (AD) [[52], [53], [54]]. Li et al. induced an inflammatory cell model using lipopolysaccharides (LPS) and measured the expression of circPTK2 (hsa_circ_0008305) and PTK2 using real-time RT-PCR [55]. They also conducted a study on the interaction between high mobility group protein B1 (HMGB1) and miR-181c-5p, as well as between circPTK2 and miR-181c-5p, using bioinformatics analysis and dual-luciferase assay. The results showed that LPS induced the release of pro-inflammatory cytokines, upregulation of HMGB1 and circPTK2, and downregulation of miR-181c-5p in microglial cells. Furthermore, miR-181c-5p was identified as a target of circPTK2 and was found to bind to HMGB1. CircPTK2 regulates LPS-induced microglial cell apoptosis by inhibiting miR-181c-5p.

Cecal ligation and puncture (CLP) was used to induce a septic mouse model, and the Morris water maze experiment and mitochondrial membrane potential (MMP) detection were used to show that silencing circPTK2 could improve cognitive function, restore MMP levels, inhibit cell apoptosis, and increase survival rates in CLP mice. He et al. reported that ethyl vanillin oxime vanadium could inhibit the cytokine signaling transduction suppressor of cytokine signaling 1/Janus kinase 2/signal transduction and activator of transcription 3 (SOCS-1/JAK2/STAT3) signaling pathway and block the cascade reaction of amyloidosis in AD mouse models, thereby reducing Aβ-induced insulin resistance in AD models [56]. Similarly, Wang et al. constructed an oxygen glucose deprivation (OGD) in vitro brain ischemia model and studied the relationship between miR-29b and circPTK2 using bioinformatics analysis, real-time RT-PCR, and luciferase analysis, as well as the role of circPTK2 in small glial cell-mediated neuronal apoptosis [57]. The results showed that circPTK2 and miR-29b share a binding site, and circPTK2 can directly bind to miR-29b. CircPTK2 regulates OGD-induced small glial cell-mediated hippocampal neuronal apoptosis through the miR-29b–SOCS–1-JAK2/STAT3-IL-1β signaling pathway. From these studies, it can be inferred that circPTK2 may be involved in small glial cell activation in AD through different pathways, but further research is needed to clarify its specific activation pathways and their relationship with the occurrence and development of AD, providing new directions and ideas for clinical prevention or treatment (Table 1).

**Table 1:** The summary highlights the circRNAs that may play a role in AD.

circRNA	Targeted miRNA/gene	Experimental subject	Expression pattern	Function	References
**mmu_circRNA_34,132, mmu_circRNA_017,077, mmu-circRNA-015,216**	–	Nrf2 (−/−) and Nrf2 (+/+) mice	–	There is a possibility that these circRNAs play a role in Nrf2-mediated neuroprotection against oxidative stress	[58]
**circHECTD1**	–	tMCAO mice	Up	Activation of astrocytes through the process of macroautophagy or autophagy	[59]
**circRNA_017,963**	miR-142/TIPARP	SAMP8 mice	Down	Closely linked to the synaptic vesicle cycle process	[60]
**ciRS-7**	NF-κB	SH-SY cells	–	Decrease in the production of Aβ.	[61]
**ciRS-7**	miR-7/UBE2A	Hippocampus of AD patients	Down	Aβ clearance	[62]
**circRNA.2837**	miR-34 family	SNI rat mode	Down	Suppression of circRNA.2837 leads to the induction of autophagy	[63]
**circPTK2**	miR-29b/SOCS1	OGD-treated microglial cell	Up	Microglial activation	[64]
**circ_0000950**	miR-103	PC12 cells and cerebral cortical neurons induced by Aβ1–42	Down	miRNA sponges	[65]
**mmu_circRNA_013,636**	–	Hippocampal tissues of SAMP8 AD mice	Up	–	[66]
**mmu_circRNA_012,180**	–	Hippocampal tissues of SAMP8 AD mice	Down	–	[66]
**circHDAC9**	miR-138	Sera of AD patients and hippocampal tissues of AD mice	Down	miRNA sponges	[67]
**circRNA KIAA1586**	miR-29b, miR-101, miR-15a	Four gene expression profiles of AD from the Gene Expression Omnibus (GEO) database	–	miRNA sponges	[68]
**circHOMER1**	miR-651	Cortex of AD patients	–	miRNA sponges	[69]
**circCORO1C**	miR-105	Cortex of AD patients	–	miRNA sponges	[69]
**circNF1–419**	Dynamin-1/AP2B1	Senescent cell model induced by d-galactose	Up	Interact with proteins	[70]
**circLPAR1**	miR-212–3p/ZNF217	Beta-amyloid (Aβ) 25-35-stimulated CHP-212 and IMR-32 cells	Up	Promotes Aβ25-35-induced apoptosis, inflammation, and oxidative stress	[71]
**circAXL**	miR-1306–5p	SK-N-SH cells with amyloid-β (Aβ1-42)	Up	Knockdown alleviated Aβ1-42-induced cell cytotoxicity, cell apoptosis, inflammation, oxidative stress and endoplasmic reticulum (ER) stress	[72]
**hsa_circ_0004381**	miR-185–5p/RAC1	MPP + -triggered SK-N-SH cells	Down	Promoted cell viability, and repressed apoptosis, inflammatory response, and oxidative stress	[73]
**circ_0005835**	miR-576–3p	BV2 cells	Up	Promoted neural stem cells (NSC) proliferation and differentiate to neuron	[74]
**circ_0000950**	miR-103	rat pheochromocytoma cell line PC12 cells and rat cerebral cortex neurons	Up	promoted neuron apoptosis, suppressed neurite outgrowth and elevated IL-1β, IL-6 and TNF-α levels	[75]
**circ_0002945**	miR-431–5p/TNFAIP1	AD serum and amyloid beta (Aβ)25-35-stimulated SK-N-SH cells and human primary neurons (HPNs)	Up	Attenuated Aβ25-35-induced cell apoptosis and endoplasmic reticulum stress	[76]
**circ_0049,472**	miR-107/KIF1B	Amyloid beta (Aβ)-induced SK-N-SH and CHP-212 cells	Up	Promoted cell proliferation, and inhibited cell apoptosis	[77]
**circ_0003611**	miR-383–5p/KIF1B	SH-SY5Y and SK-N-SH cells treated with Aβ	Up	Aβ-mediated cell proliferation, apoptosis, inflammatory response, oxidative stress, and glycolysis were abolished	[78]

### 2.6 Other circRNAs' impact on AD

There are many other circRNAs that participate in the occurrence and development of AD through different pathways. For example, Lo et al. found that the entorhinal cortex is the region with the most abundant circRNA expression, while the parahippocampal gyrus is the region with the closest correlation between circRNA and AD severity [25]. The module that is negatively correlated with AD severity in the parahippocampal gyrus is enriched in cognitive impairment and pathology-related pathways. Liu et al. found that miR-574–5p in the peripheral blood of AD patients may be a potential miRNA target of circRNA hsa_circ_0003391, participating in the occurrence and development of AD [79]. Zhang et al. confirmed through the construction of a circRNA-ceRNA network that the novel_circ_0003012/mmu-miR-298–3p/Smoc2 signaling axis may regulate the pathological and physiological processes of AD by affecting the cGMP-PKG signaling pathway [80].

**Fig. 3:**
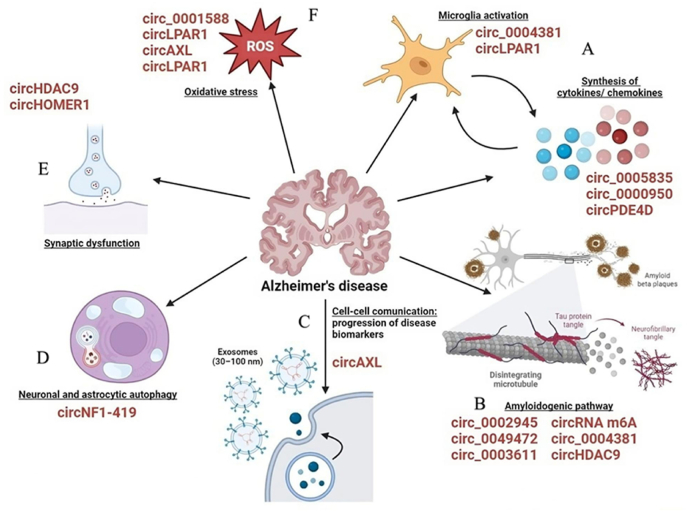
Illustrates the potential roles of circRNAs in the pathogenesis of Alzheimer's disease (AD). These circular RNAs contribute to AD development through various mechanisms: A) CircRNAs play a role in regulating microglial activation, a crucial process in neuroinflammation. They can modulate the activation state of microglial cells, impacting the inflammatory response in the brain during AD. B) They also have an impact on the production and clearance of Aβ, the key component of amyloid plaques in AD. CircRNAs can influence the levels of Aβ by regulating genes involved in its production and clearance pathways. C) Moreover, circRNAs hold promise as potential biomarkers for AD diagnosis. They are stable in plasma and enriched in exosomes, making them attractive candidates for non-invasive diagnostic tests. D) CircRNAs are involved in regulating neuronal and astrocyte autophagy, a cellular process responsible for maintaining cellular health by degrading damaged components. Dysregulation of autophagy can lead to the accumulation of toxic proteins, contributing to AD pathology. E) Additionally, circRNAs are implicated in the regulation of synaptic function. Synaptic dysfunction is a hallmark of AD and contributes to cognitive decline. CircRNAs can influence the expression of genes involved in synaptic transmission and plasticity. F) Oxidative stress is a key contributor to AD pathogenesis, and circRNAs are known to be involved in regulating oxidative stress responses in brain cells.

Li et al. found that circular RNA PTK receptor gene (circAXL), circular RNA gephyrin gene (circGPHN), and circular RNA inositol 1,4,5-trisphosphate receptor type 3 (circ-ITPR3) are independent risk factors for AD based on the analysis of the circRNA expression profile in the cerebrospinal fluid of AD patients [81]. They may have clinical value in predicting the risk and progression of AD. Wu et al. demonstrated that circular RNA recombinant lysophosphatidic acid receptor 1 (circRNA LPAR1) promotes neuronal apoptosis, inflammation, and oxidative stress induced by Aβ25-35 via the miR-212–3p/zinc finger protein 217 (ZNF217) axis, contributing to the development of AD (Fig. 3) [82].

## 3 Conclusion

In the intricate landscape of Alzheimer's disease (AD) development, circRNAs have emerged as promising candidates with significant diagnostic potential [[83], [84], [85], [86]]. To advance our understanding, future research must engage in larger-scale studies involving AD patients. These investigations should aim to pinpoint the precise stages of the disease and identify specific brain regions and cell types that undergo characteristic changes in the context of AD. A comprehensive approach will allow researchers to explore the intricate relationship between circRNA alterations and the physiological and pathological progression of AD. This holistic understanding may unveil previously unknown facets of the disease and potentially lead to the discovery of novel biomarkers, both for preclinical and clinical AD.

The quest for reliable biomarkers is pivotal for early detection, intervention, and continuous monitoring of Alzheimer's disease. By delving into the complex realm of circRNAs, researchers may unlock valuable tools for the early diagnosis and treatment of AD.

This, in turn, could pave the way for innovative approaches and targets to effectively combat this formidable neurodegenerative disorder. Considering the growing aging population and the escalating burden of neurodegenerative diseases like AD, in-depth circRNA research could have profound implications for public health. Ultimately, advancing our comprehension of circRNAs and their intricate association with AD progression holds the promise of improving patient outcomes and potentially delaying or even preventing the onset of this devastating condition. While the circRNAs discussed in this article have demonstrated their ability to act as miRNA sponges, affecting Aβ production, metabolism, autophagy, and neuroinflammatory pathways, it's important to note that circRNAs may also function through other mechanisms in AD pathogenesis, such as serving as templates for protein translation, interacting with proteins to form circRNPs, or acting as mRNA traps [87]. These alternative functions warrant further exploration. As highlighted, circRNAs play indispensable roles in AD and may hold therapeutic potential for this disease. To fully harness their potential, future studies should delve deeper into the characterization of the expression profiles and functions of additional circRNAs. This pursuit will pave the way for the development of novel therapeutic targets and biomarkers for AD. Early diagnosis of AD remains challenging, yet the timely detection of the disease offers opportunities for early intervention to potentially slow or mitigate its pathological progression. Therefore, the identification of reliable and effective biomarkers for the early stages of AD is a strategic imperative. CircRNAs that exist in serum, plasma, and cerebrospinal fluid (CSF) present themselves as strong candidates for use as diagnostic biomarkers, given their ease of identification through simple detection methods and their remarkable stability during storage and handling [88]. Future research should prioritize the use of highly sensitive RNA analysis methods to validate the utility of specific circRNAs as preclinical or clinical diagnostic biomarkers for AD. For example, one study investigated circRNA expression patterns using samples from AD patients and healthy individuals. Among the differentially expressed circRNAs, circ_0001535 emerged as a key diagnostic marker with significant potential for AD diagnosis [89]. The research identified a potential regulatory mechanism involving circ_0001535, E2F1 transcription factor, and dihydrofolate reductase (DHFR). E2F1 was found to interact with the promoter region of DHFR and to be regulated by circ_0001535, suggesting a complex interaction between circRNAs, transcription factors, and downstream effectors. The study paves the way for future research into the development of therapeutic interventions for AD, focusing on circRNAs and their interactions with key regulatory factors [89]. Understanding the role of specific circRNAs, such as circ_0001535, could provide innovative avenues for treatment strategies targeting the E2F1/DHFR axis. This research offers a valuable contribution to our understanding of AD and the potential role of circRNAs in its pathogenesis. The identification of circRNAs as diagnostic markers and their involvement in modulating critical factors like E2F1 and DHFR suggest that they may hold the key to future therapeutic strategies for AD. While further research is required to elucidate the full extent of circRNA involvement in AD, these findings represent a significant step forward in the quest to combat this devastating neurodegenerative disease.

Furthermore, circRNAs exhibit therapeutic potential, particularly in the preclinical stages of AD. Multiple studies have established that the dysregulation of ncRNAs in animal models plays a critical role in the onset and progression of AD. CircRNAs, with their ability to regulate downstream target mRNAs, have the potential to interfere with the pathological processes of AD. Notably, circRNAs could be considered as the earliest feasible pharmacological targets in AD. Some studies have already demonstrated that the use of certain miRNAs as drug targets can alleviate or treat AD in murine models. Therefore, circRNAs represent promising molecules in the landscape of AD therapy. Given their direct regulation of protein expression, synthetic circRNAs may be designed to impede the synthesis of AD-related proteins, following a therapeutic strategy akin to the approaches used with miRNAs. CircRNAs, with their role as miRNA sponges, are another intriguing class of molecules that could be explored for AD therapy. Their potential to alter the expression of miRNAs that repress target mRNAs opens exciting possibilities for innovative therapeutic interventions in Alzheimer's disease.

## Funding

This work was supported by the Bashkir State Medical University Strategic Academic Leadership Program (PRIORITY-2030).

## CRediT authorship contribution statement

**Ozal Beylerli:** Supervision, Funding acquisition, Formal analysis, Data curation, Conceptualization. **Aferin Beilerli:** Writing – original draft. **Tatiana Ilyasova:** Investigation, Data curation. **Alina Shumadalova:** Writing – review & editing. **Huaizhang Shi:** Writing – review & editing. **Albert Sufianov:** Supervision.

## Declaration of competing interest

Ozal Beylerli is an editorial board member for Non-coding RNA Research and was not involved in the editorial review or the decision to publish this article. All authors declare that there are no competing interests.

## References

[1] Lane C.A., Hardy J., Schott J.M. (2018). Alzheimer's disease. Eur. J. Neurol..

[2] Jia J., Xu J., Liu J. (2021). Comprehensive management of daily living activities, behavioral and psychological symptoms, and cognitive function in patients with Alzheimer's disease: a Chinese consensus on the comprehensive management of Alzheimer's disease. Neurosci. Bull..

[3] Barrett S.P., Salzman J. (2016). Circular RNAs: analysis, expression and potential functions. Development.

[4] Xu X., Zhang J., Tian Y. (2020). CircRNA inhibits DNA damage repair by interacting with host gene. Mol. Cancer.

[5] Shi Y., Jia X., Xu J. (2020). The new function of circRNA: translation. Clin. Transl. Oncol..

[6] Beylerli O., Gareev I., Sufianov A., Ilyasova T., Guang Y. (2022 Feb 25). Long noncoding RNAs as promising biomarkers in cancer. Noncoding RNA Res.

[7] Sufianov A., Begliarzade S., Beilerli A., Liang Y., Ilyasova T., Beylerli O. (2022 Nov 7). Circular RNAs as biomarkers for lung cancer. Noncoding RNA Res.

[8] Zhou W.Y., Cai Z.R., Liu J. (2020). Circular RNA: metabolism, functions and interactions with proteins. Mol. Cancer.

[9] Akhter R. (2018). Circular RNA and Alzheimer's disease. Adv. Exp. Med. Biol..

[10] Sufianov A., Kostin A., Begliarzade S., Kudriashov V., Ilyasova T., Liang Y., Mukhamedzyanov A., Beylerli O. (2023 Feb 7). Exosomal non coding RNAs as a novel target for diabetes mellitus and its complications. Noncoding RNA Res.

[11] Gareev I., Kudriashov V., Sufianov A., Begliarzade S., Ilyasova T., Liang Y., Beylerli O. (2022 Sep 6). The role of long non-coding RNA ANRIL in the development of atherosclerosis. Noncoding RNA Res.

[12] Markus H.S., Mäkelä K.M., Bevan S. (2013). Evidence HDAC9 genetic variant associated with ischemic stroke increases risk via promoting carotid atherosclerosis. Stroke.

[13] Lu Y., Tan L., Wang X. (2019). Circular HDAC9/microRNA-138/sirtuin-1 pathway mediates synaptic and amyloid precursor protein processing deficits in Alzheimer's disease. Neurosci. Bull..

[14] Zhong L., Yan J., Li H. (2021). HDAC9 silencing exerts neuroprotection against ischemic brain injury via miR-20a-dependent downregulation of neuroD1. Front. Cell. Neurosci..

[15] Ning Y., Ding J., Sun X. (2020). HDAC9 deficiency promotes tumor progression by decreasing the CD8+dendritic cell infiltration of the tumor microenvironment[J/OL]. J Immunother Cancer.

[16] Zhang N., Gao Y., Yu S. (2020). Berberine attenuates Aβ42-induced neuronal damage through regulating circHDAC9/miR-142-5p axis in human neuronal cells. Life Sci..

[17] Clifton N.E., Trent S., Thomas K.L. (2019). Regulation and function of activity-dependent homer in synaptic plasticity. Mol. Neuropsychiatry.

[18] You X., Vlatkovic I., Babic A. (2015). Neural circular RNAs are derived from synaptic genes and regulated by development and plasticity. Nat. Neurosci..

[19] Zhao M., Dong G., Meng Q. (2020). Circ-HOMER1 enhances the inhibition of miR-1322 on CXCL6 to regulate the growth and aggressiveness of hepatocellular carcinoma cells. J. Cell. Biochem..

[20] Li M.X., Li Q., Sun X.J. (2019). Increased Homer1-mGluR5mediates chronic stress-induced depressive-like behaviors and glutamatergic dysregulation via activation of PERK-eIF2α. Prog. Neuro-Psychopharmacol. Biol. Psychiatry.

[21] Hafez A.K., Zimmerman A.J., Papageorgiou G. (2022). A bidirectional competitive interaction between circHomer1 and Homer1b within the orbitofrontal cortex regulates reversal learning. Cell Rep..

[22] Zimmerman A.J., Hafez A.K., Amoah S.K. (2020). A psychiatric disease-related circular RNA controls synaptic gene expression and cognition. Mol. Psychiatr..

[23] Dube U., Del-Aguila J.L., Li Z. (2019). An atlas of cortical circular RNA expression in Alzheimer disease brains demonstrates clinical and pathological associations. Nat. Neurosci..

[24] Cervera-Carles L., Dols-Icardo O., Molina-Porcel L. (2020). Assessing circular RNAs in Alzheimer's disease and frontotemporal lobar degeneration. Neurobiol. Aging.

[25] Lo I., Hill J., Vilhjálmsson B.J. (2020). Linking the association between circRNAs and Alzheimer's disease progression by multi-tissue circular RNA characterization. RNA Biol..

[26] Li J., Sun Q., Zhu S. (2020). Knockdown of circHomer1 ameliorates METH-induced neuronal injury through inhibiting Bbc3 expression. Neurosci. Lett..

[27] Urdánoz-Casado A., Sánchez-Ruiz de Gordoa J., Robles M. (2021). Gender-dependent deregulation of linear and circular RNA variants of HOMER1 in the entorhinal cortex of Alzheimer's disease. Int. J. Mol. Sci..

[28] Song C., Zhang Y., Huang W. (2022). Circular RNA Cwc27 contributes to Alzheimer's disease pathogenesis by repressing Pur-α activity. Cell Death Differ..

[29] Daniel D.C., Johnson E.M. (2018). PURA, the gene encoding Pur-alpha, member of an ancient nucleic acid-binding protein family with mammalian neurological functions. Gene.

[30] Barbe M.F., Krueger J.J., Loomis R. (2016). Memory deficits, gait ataxia and neuronal loss in the hippocampus and cerebellum in mice that are heterozygous for Pur-alpha. Neuroscience.

[31] Chen J., Zou Q., Lv D. (2019). Comprehensive transcriptional profiling of porcine brain aging. Gene.

[32] Mukhopadhyay S., Jackson P.K. (2011). The tubby family proteins. Genome Biol..

[33] Rybak-Wolf A., Stottmeister C., Glažar P. (2015). Circular RNAs in the mammalian brain are highly abundant, conserved, and dynamically expressed. Mol. Cell.

[34] Dong K., He X., Su H. (2020). Genomic analysis of circular RNAs in heart. BMC Med. Genom..

[35] Wu L., Xiong L., Li J. (2020). Circ-Tulp4 promotes β-cell adaptation to lipotoxicity by regulating soat1 expression. J. Mol. Endocrinol..

[36] Chen X.J., Zhang Z.C., Wang X.Y. (2020). The circular RNome of developmental retina in mice. Mol. Ther. Nucleic Acids.

[37] Ma N., Pan J., Wen Y. (2021). circTulp4 functions in Alzheimer's disease pathogenesis by regulating its parental gene, Tulp4. Mol. Ther..

[38] Schwartzentruber J., Cooper S., Liu J.Z. (2021). Genome-wide meta-analysis, fine-mapping and integrative prioritization implicate new Alzheimer's disease risk genes. Nat. Genet..

[39] Yang Z., Jin J., Chang T. (2021). CircPTK2 (hsa_circ_0003221) contributes to laryngeal squamous cell carcinoma by the miR-1278/YAP1 axis. JAMA Oncol..

[40] Xu Z.Q., Yang M.G., Liu H.J. (2018). Circular RNA hsa_circ_0003221 (circPTK2) promotes the proliferation and migration of bladder cancer cells. J. Cell. Biochem..

[41] Wu S.G., Zhou P., Chen J.X. (2021). circ-PTK2 (hsa_circ_0008305)regulates the pathogenic processes of ovarian cancer via miR639 and FOXC1 regulatory cascade. Cancer Cell Int..

[42] Wang L., Tong X., Zhou Z. (2018). Circular RNA hsa_circ_0008305 (circPTK2) inhibits TGF-β-induced epithelial-mesenchymal transition and metastasis by controlling TIF1γ in non-small cell lung cancer. Mol. Cancer.

[43] Gong T.T., Sun F.Z., Chen J.Y. (2020). The circular RNA circPTK2 inhibits EMT in hepatocellular carcinoma by acting as a ceRNA and sponging miR-92a to upregulate E-cadherin. Eur. Rev. Med. Pharmacol. Sci..

[44] Yang H., Li X., Meng Q. (2020). CircPTK2 (hsa_circ_0005273) as a novel therapeutic target for metastatic colorectal cancer. Mol. Cancer.

[45] Yi L., Zhou L., Luo J. (2021). Circ-PTK2 promotes the proliferation and suppressed the apoptosis of acute myeloid leukemia cells through targeting miR-330-5p/FOXM1 axis. Blood Cells Mol. Dis..

[46] Zhou F., Wang D., Zhou N. (2021). Circular RNA protein tyrosine kinase 2 promotes cell proliferation, migration and suppresses apoptosis via activating microRNA-638 mediated MEK/ERK, WNT/β -catenin signaling pathways in multiple myeloma. Front. Oncol..

[47] Fan H.N., Zhao X.Y., Liang R. (2021). CircPTK2 inhibits the tumorigenesis and metastasis of gastric cancer by sponging miR-134-5p and activating CELF2/PTEN signaling. Pathol. Res. Pract..

[48] Ding Z., Sun D., Han J. (2021). Novel noncoding RNA CircPTK2 regulates lipolysis and adipogenesis in cachexia. Mol. Metabol..

[49] Chen W., Wang N., Lian M. (2021). CircRNA circPTK2 might suppress cancer cell invasion and migration of glioblastoma by inhibiting miR-23a maturation. Neuropsychiatric Dis. Treat..

[50] Ahmad M.H., Fatima M., Mondal A.C. (2019). Influence of microglia and astrocyte activation in the neuroinflammatory pathogenesis of Alzheimer's disease: rational insights for the therapeutic approaches. J. Clin. Neurosci..

[51] Katsumoto A., Takeuchi H., Takahashi K. (2018). Microglia in alzheimer's disease: risk factors and inflammation. Front. Neurol..

[52] Wang H., Lu B., Chen J. (2019). Knockdown of lncRNA SNHG1 attenuated Aβ25-35-inudced neuronal injury via regulating KREMEN1 by acting as a ceRNA of miR-137 in neuronal cells. Biochem. Biophys. Res. Commun..

[53] Yan Y., Yan H., Teng Y. (2020). Long non-coding RNA 00507/miRNA-181c-5p/TTBK1/MAPT axis regulates tau hyperphosphorylation in Alzheimer's disease[J/OL]. J. Gene Med..

[54] Souza V.C., Morais G.S., Henriques A.D. (2020). Whole-blood levels of microrna-9 are decreased in patients with late-onset Alzheimer disease. Am J Alzheimers Dis Other Demen.

[55] Li M., Hu J., Peng Y. (2021). CircPTK2-miR-181c-5p-HMGB1: a new regulatory pathway for microglia activation and hippocampal neuronal apoptosis induced by sepsis. Mol. Med..

[56] He Z., Han S., Wu C. (2020). Bis(ethylmaltolato)oxidovanadium (iv) inhibited the pathogenesis of Alzheimer's disease in triple transgenic model mice. Metallomics.

[57] Wang H., Li Z., Gao J. (2019). Circular RNA circPTK2 regulates oxygen-glucose deprivation-activated microglia-induced hippocampal neuronal apoptosis via miR-29b-SOCS-1-JAK2/STAT3-IL-1β signaling. Int. J. Biol. Macromol..

[58] Yang J.H., Zhang R.J., Lin J.J., Cao M.C., Wang Q., Cui H.X., Sun S.G., Wang L. (2018). The differentially expressed circular RNAs in the Substantia Nigra and Corpus striatum of Nrf2-Knockout mice. Cell. Physiol. Biochem..

[59] Han B., Zhang Y., Zhang Y.H., Bai Y., Chen X.F., Huang R.R., Wu F.F., Leng S., Chao J., Zhang J.H., Hu G., Yao H.H. (2018). Novel insight into circular RNA HECTD1 in astrocyte activation via autophagy by targeting MIR142-TIPARP: implications for cerebral ischemic stroke. Autophagy.

[60] Huang J.L., Qin M.C., Zhou Y., Xu Z.H., Yang S.M., Zhang F., Zhong J., Liang M.K., Chen B., Zhang W.Y., Wu D.P., Zhong Z.G. (2018). Comprehensive analysis of differentially expressed profiles of Alzheimer's disease associated circular RNAs in an Alzheimer's disease mouse model. Aging-Us.

[61] Shi Z., Chen T., Yao Q., Zheng L., Zhang Z., Wang J., Hu Z., Cui H., Han Y., Han X., Zhang K., Hong W. (2017). The circular RNA ciRS-7 promotes APP and BACE1 degradation in an NF-kappaB-dependent manner. FEBS J..

[62] Lukiw W., Zhao Y.H., Rogaev E., Bhattacharjee S. (2016). A Circular RNA (circRNA) ciRS-7 in Alzheimer's disease (AD) targets miRNA-7 trafficking and promotes deficits in the expression of the ubiquitin conjugase (UBE2A) and the epidermal growth factor receptor (EGFR). Faseb. J..

[63] Zhou Z.B., Niu Y.L., Huang G.X., Lu J.J., Chen A., Zhu L. (2018). Silencing of circRNA.2837 plays a protective role in sciatic nerve injury by sponging the miR-34 family via regulating neuronal autophagy. Mol. Ther. Nucleic Acids.

[64] Wang H., He P.H., Pan H.H., Long J., Wang J.R., Li Z.M., Liu H., Jiang W.Y., Zheng Z.M. (2018). Circular RNA circ-4099 is induced by TNF-alpha and regulates ECM synthesis by blocking miR-616-5p inhibition of Sox9 in intervertebral disc degeneration. Exp. Mol. Med..

[65] Yang H., Wang H., Shang H., Chen X., Yang S., Qu Y. (2019). Circular RNA circ_0000950 promotes neuron apoptosis, suppresses neurite outgrowth and elevates inflammatory cytokines levels via directly sponging miR-103 in Alzheimer's disease. Cell Cycle.

[66] Huang J.L., Xu Z.H., Yang S.M., Yu C., Zhang F., Qin M.C. (2018). Identification of differentially expressed profiles of Alzheimer's disease associated circular RNAs in a Panax Notoginseng Saponins-treated Alzheimer's disease mouse model. Comput. Struct. Biotechnol. J..

[67] Lu Y., Tan L., Wang X. (2019). Circular HDAC9/microRNA-138/Sirtuin-1 pathway mediates synaptic and amyloid precursor protein processing deficits in Alzheimer's disease. Neurosci. Bull..

[68] Zhang Y., Yu F., Bao S., Sun J. (2019). Systematic characterization of circular RNA-associated CeRNA network identified novel circRNA biomarkers in Alzheimer's disease. Front. Bioeng. Biotechnol..

[69] Dube U., Del-Aguila J.L., Li Z., Budde J.P., Jiang S., Hsu S. (2019). An atlas of cortical circular RNA expression in Alzheimer disease brains demonstrates clinical and pathological associations. Nat. Neurosci..

[70] Diling C., Yinrui G., Longkai Q., Xiaocui T., Yadi L., Xin Y. (2019). Circular RNA NF1-419 enhances autophagy to ameliorate senile dementia by binding Dynamin-1 and adaptor protein 2 B1 in AD-like mice. Aging (Albany NY).

[71] Wu L., Du Q., Wu C. (2021 Nov 1). CircLPAR1/miR-212-3p/ZNF217 feedback loop promotes amyloid β-induced neuronal injury in Alzheimer's Disease. Brain Res..

[72] Meng S., Wang B., Li W. (2022 Jun). CircAXL knockdown alleviates aβ1-42-induced neurotoxicity in Alzheimer's disease via repressing PDE4A by releasing miR-1306-5p. Neurochem. Res..

[73] Zhang H., Wang C., Zhang X. (2022 Aug). Circular RNA hsa_circ_0004381 promotes neuronal injury in Parkinson's disease cell model by miR-185-5p/RAC1 Axis. Neurotox. Res..

[74] Xu X., Gu D., Xu B., Yang C., Wang L. (2022 May). Circular RNA circ_0005835 promotes promoted neural stem cells proliferation and differentiate to neuron and inhibits inflammatory cytokines levels through miR-576-3p in Alzheimer's disease. Environ. Sci. Pollut. Res. Int..

[75] Yang H., Wang H., Shang H., Chen X., Yang S., Qu Y., Ding J., Li X. (2019 Sep). Circular RNA circ_0000950 promotes neuron apoptosis, suppresses neurite outgrowth and elevates inflammatory cytokines levels via directly sponging miR-103 in Alzheimer's disease. Cell Cycle.

[76] Li G., Liang R., Lian Y., Zhou Y. (2022 Jun 15). Circ_0002945 functions as a competing endogenous RNA to promote Aβ25-35-induced endoplasmic reticulum stress and apoptosis in SK-N-SH cells and human primary neurons. Brain Res..

[77] Zeng C., Xing H., Chen M., Chen L., Li P., Wu X., Li L. (2022 Sep). Circ_0049472 regulates the damage of Aβ-induced SK-N-SH and CHP-212 cells by mediating the miR-107/KIF1B axis. Exp. Brain Res..

[78] Li Y., Wang H., Chen L., Wei K., Liu Y., Han Y., Xia X. (2022 Dec). Circ_0003611 regulates apoptosis and oxidative stress injury of Alzheimer's disease via miR-383-5p/KIF1B axis. Metab. Brain Dis..

[79] Liu L., Chen X., Chen Y.H. (2020). Identification of circular RNA hsa_Circ_0003391 in peripheral blood is potentially associated with Alzheimer's disease. Front. Aging Neurosci..

[80] Zhang Y., Qian L., Liu Y. (2021). CircRNA-ceRNA network revealing the potential regulatory roles of circRNA in Alzheimer's disease involved the cGMP-PKG signal pathway. Front. Mol. Neurosci..

[81] Li Y., Fan H., Sun J. (2020). Circular RNA expression profile of Alzheimer's disease and its clinical significance as biomarkers for the disease risk and progression. Int. J. Biochem. Cell Biol..

[82] Wu L., Du Q., Wu C. (2021). CircLPAR1/miR-212-3p/ZNF217feedback loop promotes amyloid β-induced neuronal injury in Alzheimer's disease. Brain Res..

[83] Sufianov A., Begliarzade S., Ilyasova T., Liang Y., Beylerli O. (2022 Jul 6). MicroRNAs as prognostic markers and therapeutic targets in gliomas. Noncoding RNA Res.

[84] Sufianov A., Begliarzade S., Ilyasova T., Xu X., Beylerli O. (2022 Sep 22). MicroRNAs as potential diagnostic markers of glial brain tumors. Noncoding RNA Res.

[85] Beilerli A., Begliarzade S., Sufianov A., Ilyasova T., Liang Y., Beylerli O. (2022 Jul 31). Circulating ciRS-7 as a potential non-invasive biomarker for epithelial ovarian cancer: an investigative study. Noncoding RNA Res.

[86] Sufianov A., Begliarzade S., Kudriashov V., Beilerli A., Ilyasova T., Liang Y., Beylerli O. (2022 Nov 22). The role of circular RNAs in the pathophysiology of oral squamous cell carcinoma. Noncoding RNA Res.

[87] Akhter R. (2018). Circular RNA and Alzheimer's disease. Adv. Exp. Med. Biol..

[88] Puri S., Hu J., Sun Z., Lin M., Stein T.D., Farrer L.A., Wolozin B., Zhang X. (2023 Aug). Identification of circRNAs linked to Alzheimer's disease and related dementias. Alzheimers Dement.

[89] Ma M., Xie D., Zhao J. (2023 Jul 26). Bioinformatics and experimental identification of circ_0001535 associated with diagnosis and development of Alzheimer's disease. J. Integr. Neurosci..

